# Characterization of Waterborne Outbreak–associated *Campylobacter jejuni,* Walkerton, Ontario

**DOI:** 10.3201/eid0910.020584

**Published:** 2003-10

**Authors:** Clifford G. Clark, Lawrence Price, Rafiq Ahmed, David L. Woodward, Pasquale L. Melito, Frank G. Rodgers, Frances Jamieson, Bruce Ciebin, Aimin Li, Andrea Ellis

**Affiliations:** * Health Canada, Winnipeg, Manitoba, Canada; † Ontario Ministry of Health, Toronto, Ontario, Canada; ‡ Health Canada, Guelph, Ontario, Canada

## Abstract

The Walkerton, Canada, waterborne outbreak of 2000 resulted from entry of *Escherichia coli* O157:H7 and *Campylobacter* spp. from neighboring farms into the town water supply. Isolates of *Campylobacter jejuni* and *Campylobacter coli* obtained from outbreak investigations were characterized by phenotypic and genotypic methods, including heat-stable and heat-labile serotyping, phage typing, biotyping, fla–restriction fragment length polymorphism (RFLP) typing, and pulsed-field gel electrophoresis. Two main outbreak strains were identified on the basis of heat-stable serotyping and fla-RFLP typing. These strains produced a limited number of types when tested by other methods. Isolates with types indistinguishable from, or similar to, the outbreak types were found only on one farm near the town of Walkerton, whereas cattle from other farms carried a variety of *Campylobacter* strains with different type characteristics. Results of these analyses confirmed results from epidemiologic studies and the utility of using several different typing and subtyping methods for completely characterizing bacterial populations.

An outbreak of *Campylobacter jejuni* in a farming community in southern Ontario, Canada, in 1985 resulted from contamination of well water caused by spring run-off and heavy rains (*1*). In May 2000, a second waterborne outbreak of *Escherichia coli* O157:H7 and *Campylobacter* occurred in Bruce County, Ontario. Well water serving the town of Walkerton was contaminated by surface water carrying livestock waste immediately after heavy rains (*2*,*3*). A detailed microbiologic and epidemiologic analysis of the most recent outbreak may provide insights that could help make this type of outbreak less frequent.

Most sporadic cases of campylobacteriosis are associated with preparation or consumption of poultry products (*4*). Outbreaks have been associated with consumption of unpasteurized milk or unchlorinated water (*5*). An estimated 20% of cases of illness caused by *C. jejuni* are due to vehicles of infection other than food, including water (*6*). Waterborne outbreaks of *Campylobacter* tend to occur in spring or early fall, an association attributed to seasonality of surface water contamination and infection in cattle herds (*5*). Contaminated water sources have been implicated in outbreaks involving *E. coli* O157:H7 and *Campylobacter* together in Scotland (*7*) and in New York State (*8*,*9*). The former outbreak resulted from sewage contamination of the water supply of a small village in Fife, Scotland. The latter outbreak was associated with contamination of wells at a state fair (*10*). Excrement from birds and animals, including cattle, has been shown to contaminate surface water supplies used by humans infected with *Campylobacter* (*9*).

*Campylobacter* spp. have been found to cause water-borne outbreaks worldwide; such outbreaks are a particular problem in Scandinavian countries where many people drink untreated water from streams and other sources (*11*). Untreated surface water has also been implicated in *Campylobacter* outbreaks in New Zealand (*12*,*13*), Finland (*14*), England, Wales (*15*,*16*), Australia (*17*), and the United States (*18*). In Canada, outbreaks have been rarely detected and have been associated with contamination of surface water (*19*,*20*) and consumption of unpasteurized milk (*21*).

In the United States, disease caused by *C.* *jejuni* or *C*. *coli* has been estimated to affect 7 million people annually, causing 110–511 deaths and costing $1.2–$6 billion (*22*). These organisms are responsible for 17% of all hospitalizations related to foodborne illness in the United States, and although associated with a much lower case-fatality rate than *Salmonella* spp. and *E. coli* O157:H7, they account for 5% of food-related deaths (*6*). Although the incidence of *Campylobacter* infections generally appears to be higher in industrialized than in developing nations, some evidence exists that campylobacteriosis may be important from a social and economic point of view (*23*).

Epidemiologic and microbiologic analyses were undertaken to better understand the circumstances leading to the Walkerton outbreak. *C. jejuni* was isolated from patients associated with the outbreak, and *C. jejuni* and *C. coli* were isolated from animals and animal manure on farms located near the town wells. This work summarizes the phenotypic and genotypic typing results for isolates associated with the outbreak.

## Materials and Methods

### Epidemiologic Investigations

Identification of the outbreak, definition of cases, and the results of epidemiologic descriptive and cross-sectional studies have been described (*2*,*3*). Isolates from persons who did not meet all requirements for the case definition, but who resided in southwestern Ontario and became ill during the period of the outbreak, were also sent to the National Laboratory for Enteric Pathogens (NLEP), Winnipeg, Manitoba, for further analysis. A detailed description of the epidemiologic investigations is in preparation.

### Environmental Specimens

Environmental studies related to the outbreak have been described previously (*2*,*3*). Initial investigations identified 13 livestock farms within a 4-km radius of the three wells serving the town of Walkerton. From May 30 to June 13, 2000, a minimum of five manure samples per farm were obtained and tested for human enteric pathogens. Bovine rectal swabs and manure were collected from a subset of these farms in follow-up studies on June 13. All specimens were screened for *Campylobacter* spp., and isolates were forwarded to NLEP for further testing.

### Processing of Specimens

Patient stool specimens were collected into Cary-Blair transport medium and sent to the Central Public Health Laboratory, Ministry of Health and Long-Term Care, Toronto, Ontario. Specimens from animal manure were collected aseptically in sterile bags and forwarded to the same laboratory. Stools (approximately 1 g) from both sources were added into liquid enrichment medium (LEM) or directly onto charcoal-selective medium (CSM) and incubated at 42°C in a microaerobic atmosphere (5% O_2_, 10% CO_2_, 85% N_2_) for 24 h and 48 h. Cultures in LEM were subcultured to CSM and incubated as indicated above. Isolates submitted to the NLEP were routinely cultured on Mueller-Hinton agar (Oxoid Ltd., London, England) containing 10% sheep blood and stored frozen at –70°C in glycerol peptone water. Isolates were routinely incubated at either 37°C or 42°C in a microaerobic atmosphere.

### Identification of Isolates

Colonies suspected of being *Campylobacter* were gram stained and tested for oxidase, catalase, and hippurate hydrolysis. Presumptive identification of *C. coli* was achieved by the indoxyl acetate test and by determining susceptibility to nalidixic acid (30-μg disk) and cephalothin (30-μg disk). Biotyping was performed as described by Lior (*24*). In addition to biotyping, the polymerase chain reaction–restriction fragment length polymorphism (PCR-RFLP) identification scheme described by Marshall et al. (*25*) was used to confirm species identification. Primers specific to *C. jejuni* (*25*) and to *C. coli* (*26*) were used to confirm the identity of any “hippurate-negative” *C. jejuni*. Any isolates that were hippurate-negative in the tube test but positive by PCR for the hippuricase gene and negative by PCR for the aspartokinase gene associated with *C. coli* were confirmed by retesting by both methods.

### Strain Subtyping

Heat-labile (HL) serotyping was performed by the method of Lior et al. (*27*). HS serotyping, in which passive hemagglutination was used to detect heat-stable antigens, was performed by the method of Penner and Hennessy (*28*). Phage typing of isolates was performed as described by Frost et al. (*29*). Fla-RFLP typing was performed by the method of Nachamkin et al. (*30*). Numerical type designations from 1–101 were assigned at the NLEP. PFGE was done according to the method of Ribot et al. (*31*) with *Sma*I and *Kpn*I. The isolates tested by PFGE were the first human and animal isolates to be sent to the NLEP, and testing continued until type characteristics of outbreak strains were identified and the epidemiologic designations of patients involved in the outbreak were confirmed microbiologically. After this, only biotyping, serotyping, and phage typing were used to characterize outbreak strains. Fla-RFLP typing was implemented some time after the outbreak in an attempt to determine the effectiveness of this method for subtyping outbreak strains. All isolates tested by PFGE, and a random selection of isolates not tested by PFGE, were subject to fla-RFLP analysis.

## Results

A detailed description of the epidemiologic and environmental investigations is the subject of a manuscript in preparation (A. Ellis, pers. comm.). A total of 532 human stool specimens were tested for *Campylobacter* spp. Stools from 116 persons were positive for the organism, and 11 of these were also positive for *E. coli* O157:H7. Of these 116 strains, 106 were submitted to NLEP for further analysis, along with 20 strains from southern Ontario not directly linked to the outbreak. *Campylobacter* spp. (49 isolates) obtained from animals or manure on 11 of 13 farms tested were also sent to the NLEP for further analysis (*2*,*3*). No *Campylobacter* organisms were isolated from the 57 water samples tested.

All 175 isolates were characterized, first by biotyping and serotyping, then by phage typing (Table 1). A subset of 83 isolates was further characterized by PFGE, while 115 isolates were subsequently tested by fla-RFLP typing. *C. jejuni* or *C. coli* were confirmed by using PCR for the hippuricase and aspartokinase genes, a strategy that also allowed the definitive identification of hippuricase-negative (hipp. neg.) *C. jejuni* strains. Five biotypes (I, II, III, IV, and hipp. neg.) were found among the isolates, with biotype II predominating. HS serotyping detected 14 different serotypes among the larger group of 175 isolates. Three HS serotypes were epidemiologically associated with the outbreak (Tables 1 and 2). Most outbreak-associated strains were HS serotype O:2. Phage typing was useful for further strain discrimination, yielding 22 PTs (25 if phage type variants were included) plus two isolates with atypical lytic patterns and two untypeable strains. PT 33 was most commonly associated with outbreak strains, though other phage types were also outbreak-associated. HL serotyping generated 29 types from the group of 175 strains. PFGE divided the 83 strains tested into more than 30 types when both *Sma*I and *Kpn*I were used (Tables 2 and 3). Though fla-RFLP typing produced 22 different types, only 7 were epidemiologically associated with the outbreak. When combined, the results from all phenotypic and genotypic assays created a large number of distinct types (Tables 2, 3, and 4). HL serotyping allowed typing of 150 (86%) of 175 isolates tested. HS serotyping achieved 97% typeability, while phage typing and molecular typing methods typed 99% and 100% of strains tested, respectively.

**Table 1:** Tests used for analysis of *Campylobacter* isolates from Bruce-Grey county Ontario, Spring 2000^a^

Test	No. strains tested	No. types obtained	No. types outbreak associated
Species	175	2	1
Biotype	175	5	3
HS serotype	175	14	3
Fla-RFLP type	115	22	7
Phage type	175	27	14
HL serotype	175	29	13
PFGE type (*Sma*I)	83	30	6
PFGE type (*Kpn*I)	65	17	4

^a^HS, heat-stable; RFLP, restriction fragment length polymorphism; HL, heat-labile; PFGE, pulsed-field gel electrophoresis.

**Table 2:** Characteristics of *Campylobacter jejuni* strains from human patients^a^

Species	Biotype^a^	HS type	Fla-RFLP type	PFGE type using *Sma*I	PFGE type using *Kpn*I	No. strains	Outbreak type
*Campylobacter jejuni*	II	O:2	1	CASAI.0001	CAKNI.0001	13	1
*C. jejuni*	II	O:2	1	CASAI.0001	CAKNI.0001	1	NER
*C. jejuni*	II	O:2	1	CASAI.0002	CAKNI.0002	8	1
*C. jejuni*	II	UT^a^	1	CASAI.0002	CAKNI.0002	1	1
*C. jejuni*	II	O:2	1	CASAI.0002	CAKNI.0002	2	NER
*C. jejuni*	II	O:2	1	CASAI.0002	CAKNI.0036	1	NER
*C. jejuni*	II	O:2	1	CASAI.0002	CAKNI.0003	1	1
*C. jejuni*	hipp.neg.^a^	O:2	1	CASAI.0002	CAKNI.0003	1	1
*C. jejuni*	II	O :2	1	CASAI.0004	CAKN1.0001	2	1
*C. jejuni*	II	O:2	1	CASAI.0011	CAKNI.0001	1	1
*C. jejuni*	II	O:2	1	ND^a^	ND	14	1
*C. jejuni*	II	O:2	ND	ND	ND	20	1
*C. jejuni*	II	O:2	ND	ND	ND	1	NER
*C. jejuni*	II	O:2	ND	ND	ND	28	1
*C. jejuni*	II	UT	ND	ND	ND	1	NER
*C. jejuni*	hipp. neg.	O:2	34	CASAI.0003	CAKNI.0003	9	2
*C. jejuni*	hipp. neg.	O:2	34	CASAI.0003	CAKNI.0003	1	2
*C. jejuni*	II	O1,44	2	CASAI.0012	CAKNI.0012	1	NER
*C. jejuni*	I	O:3	ND	ND	ND	1	NER
*C. jejuni*	II	O:4 complex	93	ND	ND	2	Not defined
*C. jejuni*	II	O:4 complex	93	ND	ND	1	NER
*C. jejuni*	II	O:4 complex	ND	ND	ND	1	NER
*C. jejuni*	II	O:4 complex	90	CASAI.0030	CAKNI.0024	1	NER
*C. jejuni*	I	O:4 complex	94	ND	ND	1	NER
*C. jejuni*	II	O:4 complex	90	ND	ND	1	Not defined
*C. jejuni*	II	O:17 complex	1	ND	ND	1	NER
*C. jejuni*	I	O:11	91	CASAI.0029	CAKNI.0026	1	NER
*C. jejuni*	III	O:17 complex	5	ND	ND	1	Not defined
*C. jejuni*	I	O:17 complex	99	ND	ND	1	NER
*C. jejuni*	III	O:21	5	ND	ND	1	Not defined
*C. jejuni*	IV	O:21	5	ND	ND	1	NER
*C. jejuni*	II	O:17 complex	4	ND	ND	1	Not defined
*C. jejuni*	III	O:17 complex	5	ND	ND	1	Not defined
*C. jejuni*	III	O:17 complex	5	ND	ND	1	NER
*C. coli*	I (*C. coli*)	O:34	36	CASAI.0020	CAKNI.0025	1	NER
*C. jejuni*	II	O:35	92	ND	ND	1	NER
*C. coli*	I	O:47	82	CASAI.0010	CAKNI.0004	1	NER

^a^HS, heat-stable; RFLP, restriction fragment length polymorphism; PFGE, pulsed-field gel electrophoresis; NER, not epidemiologically related to the outbreak; UT, untypeable; hipp. neg., lack of hippurate hydrolysis in *C. jejuni* strains; ND, not determined

**Table 3:** Characteristics of *Campylobacter* strains found from cattle on farms during the outbreak

Farm	Species	Biotype^a^	HS serotype	Fla-RFLP type	PFGE type using *Sma*I	PFGE type using *Kpn*I	No. strains	Outbreak associated
1	*C. jejuni*	II	O:1	33	CASAI.0034	ND^a^	1	No
	*C. jejuni*	II	UT^a^	33	CASAI.0034	ND	1	No
	*C. jejuni*	II	O:4 complex	90	CASAI.0026	ND	1	No
	*C. jejuni*	II	O:4 complex	90	CASAI.0030	CAKNI.0024	1	No
	*C. jejuni*	II	O:4 complex	ND	ND	ND	1	No
	*C. jejuni*	I	O:18	ND	ND	ND	1	No
	*C. jejuni*	I	O:18,37	33	CASAI.0013	ND	1	No
	*C. coli*	I	O:26,30,34	ND	ND	ND	1	No
	*C. coli*	I	O:34	ND	ND	ND	1	No
	*C. jejuni*	II	O:35	2	CASAI.0033	ND	1	No
	*C. jejuni*	II	O:35	ND	ND	ND	1	No
2	*C. jejuni*	II	O:2	1	CASAI.0001	CAKNI.0001	1	Yes
	*C. jejuni*	II	O:2	1	CASAI.0004	CAKNI.0001	2	Yes
	*C. jejuni*	II	O:2	1	CASAI.0004	CAKNI.0005	1	Yes
	*C. jejuni*	II	O:2	1	ND	ND	3	Yes
	*C. jejuni*	II	O:2	ND	ND	ND	2	Yes
	*C. jejuni*	II	O:2	1	ND	ND	2	Yes
	*C. jejuni*	hipp. neg.	O:2	34	CASAI.0003	CAKNI.0003	2	Yes
3	*C. jejuni*	III	O:38	74	CASAI.0005	CAKNI.0006	1	No
	*C. jejuni*	III	O:4 complex	95	CASAI.0006	CAKNI.0007	1	No
	*C. jejuni*	III	O:13,50,65	95	CASAI.0006	CAKNI.0007	1	No
5	*C. jejuni*	II	O:1	97	CASAI.0007	CAKNI.0008	1	No
6	*C. jejuni*	II	O:4 complex	6	CASAI.0017	ND	1	No
	*C. jejuni*	II	O:18	33	CASAI.0014	ND	1	No
7	*C. jejuni*	II	O:4 complex	93	CASAI.0016	ND	1	No
	*C. jejuni*	II	O:4 complex	93	ND	ND	1	No
	*C. jejuni*	II	O:4 complex	90	ND	ND	1	No
	*C. jejuni*	II	O:13	93	CASAI.0007	CAKNI.0009	1	No
	*C. jejuni*	II	O:13,O64	93	CASAI.0015	CAKNI.0028	1	No
8	*C. coli*	I	O:25	73	CASAI.0019	ND	2	No
	*C. coli*	I	O:34	98	CASAI.0018	ND	1	No
9	*C. coli*	I	O:34	36	CASAI.0020	CAKNI.0025	1	No
	*C. jejuni*	II	O:11	ND	ND	ND	1	No
	*C. jejuni*	II	UT	52	UT	ND	1	No
10	*C. jejuni*	I	O:11	91	CASAI.0031	CAKNI.0027	1	No
	*C. jejuni*	II	O:4 complex	6	CASAI.0022	CAKNI.0029	1	No
	*C. jejuni*	II	O:35	2	CASAI.0021	ND	1	No
	*C. jejuni*	II	UT	2	CASAI.0021	ND	1	No
12	*C. jejuni*	II	O:2	101	CASAI.0025	ND	1	No
	*C. coli*	I	O:34	36	CASAI.0024	ND	1	No
	*C. jejuni*	I	O:35	2	CASAI.0023	ND	1	No
14	*C. jejuni*	II	O:2	1	ND	ND	1	No

^a^HS, heat-stable; RFLP, restriction fragment length polymorphism; PFGE, pulsed field gel electrophoresis; ND, not determined; UT, untypeable.

**Table 4:** Variability of phage typing (PT) and heat-labile (HL) type in outbreak strains 1 and 2^a^

PT	HL Type		No. isolates						Total no. isolates	
			fla-RFLP and PFGE types NT		fla-RFLP type 1, PFGE types NT	fla-RFLP type 1, PFGE strain 1 types	fla-RFLP types 1 & 99, PFGE strain 2 types			
Outbreak strain type 1 (HS O:2 or UT; fla RFLP type 1 or ND^a^; PFGE types CASAI.0001, .0002, 4, 11, ND, CAKN.0001, 2, 3, ND)										
13	128		-		-	1	-			1
31	110		-		-	1	-			1
33	4		1		1	-	-			2
	[4,125]		1		2	-	-			3
	100		1		1	-	-			2
	110		1		-	-	-			1
	112		1		1	-	-			2
	[112,125]		2		2	-	-			4
	125		20		-	19	-			39
	128		9		2	1	-			12
	[125,128]		-		3	1	-			4
	UT^a^		10		-	3	-			13
33 var.	UT		-		-	1	-			1
35	125		-		-	1	-			1
40	125		1		-	-	-			1
64	128		-		1	-	-			1
UT	128		1		-	-	-			1
Outbreak strain type 2 (HS O:2; fla-RFLP type 34 or 99; PFGE types CASAI.0003, ND, CAKNI.0003, ND)										
13		128	-	-		-		2		2
		UT	-	-		-		2		2
14		UT	-	-		-		1		1
28		4	-	-		-		1		1
		100	-	-		-		1		1
71		4	-	-		-		2		2
		100	-	-		-		1		1
Total			48	13		28		10		99

^a^RFLP, restriction fragment length polymorphism; PFGE, pulsed-field gel electrophoreses; ND, not determined; UT, untypeable.

**Figure 1:**
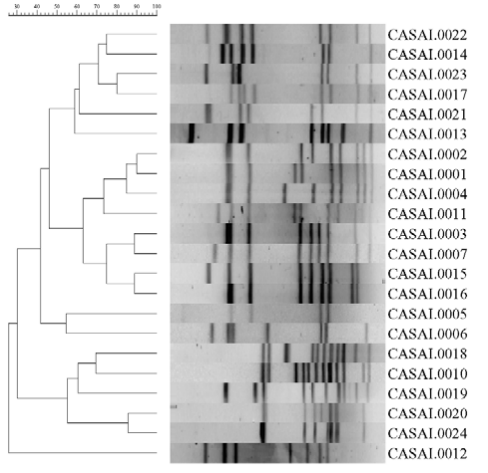
Dendrogram showing pulsed-field gel electrophoresis of *Campylobacter* isolates using *Sma*I.

**Figure 2:**
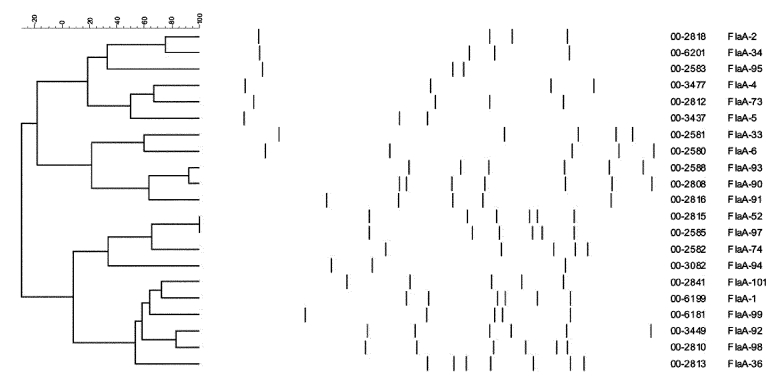
Dendrogram showing *Campylobacter* fla–reverse fragment length polymorphism types.

The characteristics of outbreak strains were derived by correlating the results of phenotypic and genotypic assays. Only biotypes II and hipp.-neg. *C. jejuni* were strongly associated with the outbreak, although 13 biotype III isolates were also identified. Most (99/106; 92%) of the patient isolates epidemiologically associated with the outbreak expressed HS serotype O:2 (Table 2). The most common phage types among all isolates tested were PT 33 (100/175 isolates), PT 13 (12/175 isolates), and PT 1 (10/175 isolates). These phage types were found in isolates epidemiologically associated with the outbreak as well as those that were not, although 82/106 (77%) of patient isolates associated with the outbreak were PT 33 (Table 2). Of the 57 fla-RFLP type 1 isolates characterized, 50 (88%) were epidemiologically associated with the outbreak. PFGE *Sma*I types CASAI.0001, .0002, .0004, and .0011 clustered on the same branch of a dendrogram constructed with PFGE patterns from isolates obtained at the time of the outbreak (Figure 1) and were closely associated with other type characteristics connected with the outbreak, including HS serotype O:2; fla type 1; PT 33; and HL types 125, 128, and UT. Five other isolates considered epidemiologically unrelated to the outbreak had Penner type O:2, fla-RFLP type 1, PT 33, and biotype II. Four of these isolates were HL serotype 125. One strain had the PFGE outbreak type CASAI.0001, CAKNI.0001, and three of the four other strains had outbreak type CASAI.0002, CAKNI.0002. The final strain had PFGE type CASAI.0002 and CAKNI.0036, a PFGE pattern varying from CAKNI.0002 only by two minor bands at the bottom of the gel. In this context, all strains were considered outbreak type 1 strains. A second fla-RFLP type, 34, was closely associated with 11 isolates from both humans and animals, all of which were epidemiologically associated with the outbreak. All strains with fla-RFLP type 34 were HS serotype O:2, hipp. neg., and PFGE type CASAI.0003, CAKNI.0003, although four different HL serotypes and five different phage types were present (Table 4). In addition, one strain with fla-RFLP type 99 was HS serotype O:2, hipp. neg., and PFGE type CASAI.0003, CAKNI.0003. This combination of types and subtypes was considered outbreak type 2 (Table 2). As shown in Figure 2, although fla-RFLP type 99 is more similar to type 1 than type 34, it still differs from type 1 by three bands. Only seven other isolates representing a few other distinct *C.* *jejuni* types were also considered to be epidemiologically associated with the outbreak (Table 2).

Farms near the town of Walkerton were considered as possible sources of bacteria causing the outbreak. Sampling of animals on 11 farms yielded a number of *C. jejuni* and *C. coli* isolates (Table 3). Isolates of outbreak type 1 were found from cattle on farm 2 and farm 14, whereas the second outbreak type was found in cattle on farm 2. A variety of different strains were obtained from other farms, although none expressed characteristics of the two major outbreak types.

Two of the strains recovered from animals on farm 7 shared some characteristics with isolates from humans epidemiologically associated with the outbreak. A patient isolate with HS serotype O:4 complex (O13), biotype II, phage type 6, fla type 91, and HL serotype 7 was similar to an animal isolate with HS serotype O:4 complex (O:13), biotype II, phage type 6, fla type 93, and HL serotype UT. Two isolates from humans associated with the outbreak had HS serotype O:4 complex, biotype II, fla type 93, HL serotype 7, and PT 13 or 71. Isolates from animals on farms 1 and 7 were similar but were considered epidemiologically unrelated to the outbreak (*2*,*3*).

### Methods Used for Strain Characterization

Many isolates were distinguishable by types obtained with only one or two methods, while all other types remained the same. Some strains varied only in the expression of their O:4 complex (O:4, O:13, O:16, O:43, and O:50 [*32*]) HS serotypes (data not shown). A single patient isolate with serotype O:2, the hipp. neg. *C. jejuni* biotype, and PFGE type CASAI.0003 differed from a group of nine other patient isolates by expressing fla type 99 rather than type 34 (Table 2, Figure 2); several HL serotypes and phage types were found within this group of isolates (Table 4). Two strains with HS serotype O:17 complex (O:17,23,36), and HL serotype 5 had different fla-RFLP types, phage types, and biotypes. Two similar bovine isolates from farm 1 had an identical fla-RFLP type (type 90) and similar HS serotypes (O:4 complex) but had different phage types, HL serotypes, and PFGE types (Table 3). All strains from farm 7 carried some combination of types that included HS serotypes O:4 complex, either fla-RFLP types 90 or 93, HL serotype 7 or UT, and a number of phage types. Most phage types did not show a 1:1 correlation with types obtained with other methods or with the outbreak (Table 4). HL serotyping appeared to be more discriminatory than the other methods used, although HL serotypes did not appear to change at random from types obtained with all other methods. The HL types associated with the outbreak were found only in isolates with HS serotype O:2 (Table 4).

## Discussion

Phenotypic and molecular typing methods together support the hypothesis that bacteria entered the Walkerton municipal water supply from neighboring farms and implicate farm 2 as the major source of outbreak strains. This conclusion was consistent with hydrogeologic models in which runoff from heavy rains swept *Campylobacter* spp. and *E. coli* O157:H7 bacteria from farm 2 into the vicinity of well 5, where they gained access to the well and were distributed through the town’s water supply (*2*,*3*). A few isolates indistinguishable from the outbreak strain were recovered from patients not epidemiologically associated with the outbreak, suggesting that these patients might indeed have been associated with the outbreak. The outbreak case definition would not exclude sporadic cases occurring at the same time as the outbreak. These isolates could represent cases of secondary transmission or patients having an indirect association with the outbreak that were not identified during the epidemiologic investigation.

Isolates from some patients who were epidemiologically associated with the outbreak produced molecular subtyping results that differed from the outbreak type, suggesting that these bacteria might have been acquired from a source other than well 5 or that they may have been present on farm 2 adjacent to well 5 but not detected. These organisms could have entered the water supply near well 6, though that well was not as susceptible to contamination as well 5 (*3*). If well 6 was involved, isolates with the types found on other farms (e.g., farm 7) near the well should have comprised a higher proportion of outbreak strains. Patients could have acquired the organisms through direct or indirect contact with animals or persons from farms or from some other common source. Strains with characteristics similar to these non-O:2 strains were often not found on farms in the Walkerton area. Though the outbreak affected many residents in this area, it may have occurred against a background of sporadic cases.

The diversity seen among the *Campylobacter* isolates is in striking contrast to the single *E. coli* type infecting Walkerton outbreak patients and in cattle on farm 2 (2,3, data not shown). Furthermore, during the New York state fair outbreak, a single *Campylobacter* PFGE type predominated (*9*). Isolates from a point source outbreak caused by tuna salad had the same HS serotype, HL serotype, and biotype (*33*). Routine surveillance of *Campylobacter* by HS serotyping and phage typing identified a single type that caused an outbreak associated with stir-fried food in the United Kingdom (*34*). HS serotype, ribotype, DNA profile, and PFGE all showed the same profile in isolates obtained from a 6-week continuous source waterborne outbreak in a town in Denmark (*35*). A damaged sewer line was implicated in this outbreak. In contrast, of 25 outbreaks investigated by Frost et al. (*36*), isolates with only one PT and HS serotype were found in 13 outbreaks and multiple types (up to eight) in 12 outbreaks. The diversity of HS serotypes and PFGE types encountered in Walkerton may therefore be somewhat unusual, while the diversity of HL serotypes and phage types is consistent with information in the literature. This diversity could be the result of inclusion of strains or types that were not outbreak related or from the heterogeneity of types at nearby farms. Existing data do not allow us to determine which of these hypotheses is correct.

Continuous, comprehensive databases of molecular subtyping data for *Campylobacter* species have not yet been developed in Canada. Whether the Walkerton outbreak types are rare types or common types in Canada is not known. This uncertainty makes interpretation of the data more difficult and highlights the need for continuous surveillance of pathogens to support the interpretation of typing and fingerprinting data.

Different methods performed quite differently for characterizing strains. Fla-RFLP typing and Penner serotyping appeared to group strains into larger clusters, which was useful for identifying outbreak-associated strains. Results from these two methods together would have allowed good predictions about whether a *Campylobacter* isolate should be included in the outbreak investigation. A close correspondence has previously been found for *fla*A RFLP types and *Sma*I PFGE types which, together with HS serotypes, were found to identify *C. coli* clonal lines having epidemiologic significance (*37*). HS serotype O:2 appears to be a common strain of *Campylobacter* (*38*) and is found frequently in isolates from both humans and cattle (*39*,*40*). Additional information from fla-RFLP typing may therefore be necessary for more definitive discrimination. Several isolates belonged to the HS O:4 complex, with each antigen expressed variably in individual strains. Strains expressing this complex predominated on farms 3 and 7 (Table 3) and were also found in three isolates from patients (Table 2). Only isolates from farm 7, near well 6, had the fla-RFLP types 90 and 93 in common with patient isolates. The sources of infection of these patients was not clear, although well 6 probably did not become contaminated (*3*).

PFGE data correlated well with HS serotyping and fla-RFLP data. A group of closely related PFGE patterns was associated with the outbreak. PFGE was more discriminatory than fla-RFLP typing and HS serotyping, and during the outbreak, additional information had to be collected to associate all five *Sma*I PFGE types with the outbreak. This limited the utility of PFGE for identifying outbreak strains until epidemiologic data were available. Although fla-RFLP typing had a lower apparent discriminatory power compared with PFGE, it was more useful for organizing strains into epidemiologically relevant groups. Close examination of PFGE patterns CASAI.0001, .0002, .0004, and .0011 indicated an underlying similarity, with changes that suggested duplication, insertion, or deletion of DNA from a common ancestral type (Figure 1). Strains with patterns CASAI.0001, .0004, and .0011 all shared a common *Kpn*I restriction pattern (CAKNI.0001). Differences in these patterns are consistent with changes within restriction sites or rearrangements. PFGE pattern CASAI.0002, however, was associated with patterns CAKNI.0002 and CASAI.0003. CAKNI.0002 differs from CAKNI.0001 only in the position of the top band, which is higher in CAKNI.0001 (data not shown). Patterns CASAI.0001, .0004, and .0011 differed from CASAI.0002 by the position of a single larger band in each pattern (Figure 1). These differences are more consistent with the addition of DNA through insertion of exogenous material or duplication of chromosomal loci. Recombination appears to occur frequently within *Campylobacter* species and, with genomic rearrangement, contributes to the genomic instability characteristic of certain strains (*39*–*41*). The events causing the PFGE changes seen in these closely related patterns remain to be determined. That such changes may occur at relatively high frequency is suggested by the discriminatory power of PFGE compared with other typing methods.

Phage typing was useful in defining the outbreak strains in early stages of the investigation due to the speed with which results could be obtained. However, the second most common outbreak type isolated, defined by HS serotype O:2, hipp. neg. biotype, fla-RFLP type 34, PFGE CASAI.0003, had several phage types (13, 14, 28, 33 var., 71). Phage types varied independently of the other characteristics measured (Table 4), giving this typing method a higher apparent discriminatory power than HS serotyping, fla typing, or PFGE. Including isolates into the outbreak on the basis of phage type alone,without accompanying epidemiologic data, would have been difficult. This factor may limit the utility of phage typing for detection of outbreaks, though at least one outbreak has been identified on the basis of phage typing and HS serotyping. PT 33 was, however, an effective marker for the most prevalent outbreak type.

HL serotypes 4, [4,125], 100, 112, [112, 125], 125, [125,128], and 128 were almost exclusively associated with outbreak isolates. Though it would have been difficult to identify outbreak strains on the basis of HL serotype alone, this method did help confirm the link between outbreak strains in humans and isolates from farm 2. The 125 and 128 serotypes have been seen infrequently by NLEP and are more “unique” markers than either HS serotype O:2 or phage type 33. Serotype HL 5 was associated with the HS O:17 complex in isolates from human patients and was not found in isolates from any of the farms. The source of these isolates was not determined, though O:17 strains have previously been recovered from poultry (*24*). HL serotype 7 was associated with the HS O:4 complex discussed earlier, suggesting that the associations between HL and HS serotypes noted previously (*23*) may not be random. Within the outbreak strain, however, changes in HL serotype appeared to occur more frequently than, and independently from, other type characteristics. HL typing would not have been of use in the identification of the two Walkerton outbreak strains if used in the absence of epidemiologic information. That two HL serotypes could be found, namely types [4, 125], [112, 125], and [125, 128], all of which included HL 125, was interesting. Further characterization of these complex HL serotypes could provide useful laboratory-based epidemiologic information.

In summary, two *Campylobacter jejuni* strains were associated with the Walkerton outbreak through the use of different typing and subtyping methods in combination with epidemiologic data. These methods were useful for defining the scope of the outbreak, for identifying the source of strains, and for tracing the route by which bacteria infected humans. The bacteriologic findings fully support the results of the epidemiologic and hydrogeologic investigations (*2*,*3*), which suggest that bacteria from cattle manure were able to enter groundwater after heavy rains and contaminate a well serving the town of Walkerton, subsequently infecting those consuming the water. Some investigators think that adult beef cattle represent a limited threat to water supplies and subsequent transmission of *Campylobacter* to humans (*42*). However, recent investigations suggest that the environment, as well as cattle and other farm animals, may play an important role in human infection with these organisms (*38*,*39*). Studies of the contribution of cattle feedlots and other farm operations to *Campylobacter* contamination of surface waters and watersheds, as well as subsequent human infections, would provide useful information for farm management practices and the protection and management of water resources.
